# Salivary Uric Acid: A Noninvasive Wonder for Clinicians?

**DOI:** 10.7759/cureus.19649

**Published:** 2021-11-16

**Authors:** Arpita Jaiswal, Sparsh Madaan, Neema Acharya, Sunil Kumar, Dhruv Talwar, Deepika Dewani

**Affiliations:** 1 Department of Obstetrics and Gynaecology, Jawaharlal Nehru Medical College, Datta Meghe Institute of Medical Sciences (Deemed to be University), Wardha, IND; 2 Department of Medicine, Jawaharlal Nehru Medical College, Datta Meghe Institute of Medical Sciences (Deemed to be University), Wardha, IND

**Keywords:** human immunodeficiency virus, hyperuricemia, cancer, saliva, uric acid

## Abstract

This review is a summary of the modern-day approach and recent trend in the determination of uric acid in the saliva of humans and its use in diagnosis by clinicians. Uric acid, which is the end product obtained from the breakdown of purine nucleotides, is an important biomarker associated with various conditions. Uric acid is found in various body fluids, such as serum, plasma, and urine. It can be used as an important tool for various diseases, such as gout and hyperuricemia, or conditions that are associated with increased oxidative stress. Recently, there has been an emergence of studies that have utilized uric acid concentrations measured in the saliva and studied its association with various diseases. Salivary uric acid can prove to be a noninvasive method to provide a diagnosis of serious illness. A raised uric acid level in the saliva can be associated with cancer, human immunodeficiency virus (HIV) infection, gout, and hypertension. A reduced level of salivary uric acid on the other hand can be a marker for Alzheimer’s disease, progression of multiple sclerosis, and impairment of cognition.

Online search databases, including Google Scholar, Scopus, PubMed, and Web of Science, were searched, and articles that were published before September 2021 based on salivary uric acid analysis were analyzed for this review.

Uric acid is an essential biomarker that has antioxidant properties. Assessment of salivary uric acid levels was found to be essential in conditions such as cancer, metabolic syndrome, neurological conditions, psychiatric conditions, human immunodeficiency virus, and gout and in monitoring treatment of hyperuricemia.

Although having importance in diagnosis and therapeutic monitoring, salivary uric acid analysis has not gained enough popularity due to limitations such as saliva collection and sample processing issues. With proper education and standardization, salivary uric acid analysis can be used as a cost-effective and noninvasive tool for getting a clue about antioxidant biomarker concentration in saliva and hence various diseases associated with oxidative stress.

## Introduction and background

Human saliva is known to be composed of 99% water along with inorganic salts that comprised sodium, potassium, calcium, phosphate, and bicarbonate, with some organic compounds that include uric acid, lactate, hormones, polypeptides, and proteins such as enzymes, mucins, and immunoglobulins [1]. Some of the other constituents of saliva that might be important as a biomarker are neopterin, nitrites, nitrates, and glutathione, which can be isolated from saliva. Studies have been conducted to determine the amount of immunoglobulin A (IgA) in the saliva of patients diagnosed with human immunodeficiency virus (HIV), and these studies have shown that anti-HIV IgA antibody isolated from the saliva may prove to be an important tool for the prognosis of patients with HIV by determining the chances of disease progression [2].

Saliva has numerous functions, including lubrication, providing local immunity, taste, buffering action, digestion, and maintaining the integrity of teeth, and saliva also has antifungal, antibacterial, and antiviral activities. Increasing research in the modern era is focusing on the use of saliva as an important diagnostic tool. Saliva collection is a safe, simple, and noninvasive procedure with a very low chance of infections when compared to other invasive methods such as the sampling of the blood where there are great challenges associated with the collection of blood in cases of children, irritable patients, and patients who are handicapped either mentally or physically. A limitation in the sampling of saliva, however, remains the compromise in diagnosis that occurs as a result of increased dilution of analytes, interference caused by food, and diseases of the tooth and in cases of smokers [3]. Uric acid is the end product formed from the metabolism of purine. Uric acid is excreted from the body primarily in the urine by the kidneys. Abnormally excessive accumulation of uric acid in the blood might lead to a condition known as gout where crystals of monosodium urate form in the joints, synovial fluid, tendons, and surrounding tissues. Besides conditions such as gout, an increase in uric acid levels can also be witnessed in conditions such as hypertension, stroke, various renal and cardiovascular disease, and metabolic syndrome [4]. The level of uric acid in a healthy individual is 199 ± 27 μmol/L, whereas the normal concentration of uric acid in the serum is 120-400 μmol/L. Various studies have reported a linear relationship between uric acid levels measured in serum and saliva [5].

A number of studies conducted recently have established the importance of salivary uric acid estimation and its potential to replace other urine and blood tests [6]. Presently, laboratories worldwide are using saliva majorly for the estimation of immunoglobulin A, cortisol, testosterone, and drugs [7]. Uric acid is presently not being estimated in saliva routinely despite its importance in the diagnosis of various medical conditions owing to its essential antioxidant action.

Enzymatic assay kits are primarily used to estimate the levels of uric acid, which may be in serum, urine, or saliva [8]. These kits are cost-effective; however, the inadequate volume of sample, decreased sensitivity, and early expiration of the reagents used might limit their usage due to false-positive or false-negative results.

Search strategy

The present comprehensive review was conducted using Preferred Reporting Items for Systematic Reviews and Meta-Analyses (PRISMA) guidelines, addressing the role of salivary uric acid and serum uric acid and their correlation with the diagnosis of various clinical conditions. Electronic and manual data resources were consulted using the following databases: PubMed/MEDLINE, Embase, Science Direct, Cochrane Library, and clinical trials site for studies published until September 2021. The results were filtered for studies that described in the English language. The words that were used for the search strategy on various databases were salivary uric acid and serum uric acid. The literature search on PubMed/MEDLINE was based on the terms “salivary uric acid” [All Fields] AND “serum uric acid” [MeSH Terms] OR “oxidative stress” [All Fields]. The abovementioned words (salivary uric acid and serum uric acid) were used in the search strategy on the Cochrane Library, the database for systematic review.

Selection criteria

**Figure 1:**
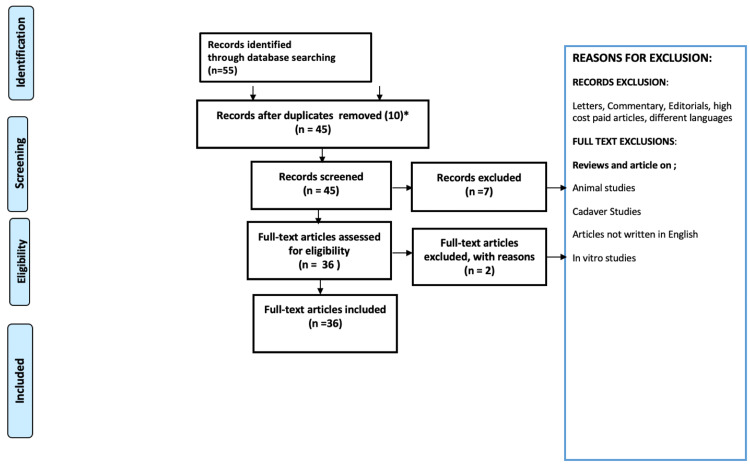
Study selection process using the Preferred Reporting Items for Systematic Reviews and Meta-Analyses (PRISMA) guidelines

The selection criteria included systematic reviews, randomized and non-randomized human controlled trials, and retrospective and prospective cohort studies. Studies composed of the correlation of salivary uric acid and serum uric acid were included. Technical reports, animal studies, cadaver studies, in vitro studies, case reports, letters to the editor, and review papers were not included (Figure 1).

Study selection

Three of the reviewers screened all identified titles and abstracts independently. In addition, the reference lists of the selected abstracts and the references of systematic reviews, randomized and non-randomized human controlled trials, and retrospective and prospective cohort studies were manually searched. The full text was accessed for studies appearing to meet the selection criteria or for which improper data in the abstract and title was available.

## Review

Serum and salivary uric acid levels in neurological and psychiatric conditions

Purines such as adenosine and adenosine triphosphate along with uric acid have been known to modulate the functions of the central nervous system, including the threshold of convulsions, cognition, memory, appetite, sleep, activity, mood, social interaction, and intelligence. A reduced level of serum uric acid level has been reported in neurodegenerative conditions, including amyotrophic lateral sclerosis, Alzheimer’s disease, and Parkinson’s disease. This suggests that uric acid might exert neuroprotective effects. Studies conducted in the past have reported an elevation of uric acid levels in the plasma of patients with bipolar disorder and reduced levels in cases of anxiety disorders and major depression [9]. The levels of uric acid normalized following treatment of the above psychiatric conditions, thus further supporting the association. Umeki reported that 11 out of a total of 27 patients who had chronic anorexia nervosa were found to have markedly raised levels of uric acid upon admission [10]. Gupta et al. found that 9% of patients presenting with a consecutive eating disorder had raised levels of uric acid upon initial presentation, which might be explained by an increase in the degeneration of ATP as a result of increased physical exertion or a decrease in uric acid clearance by the renal system due to starvation [11]. A study conducted by Giesser et al. that included the adolescent population showed that salivary uric acid levels were raised significantly in patients with eating disorders when compared with control [12]. During the reproductive years, women usually have reduced levels of uric acid when compared with men, which are in part a result of the uricosuric effect exerted by estrogen and progesterone on the uric acid clearance by the renal system. Adolescents and adults who manifest with anorexia nervosa have reduced levels of estradiol in their serum, which may result in an increase in the uric acid levels along with other abnormalities such as menstrual dysfunction. This postulate is supported by the fact that hormonal therapy is successful in suppressing uric acid levels and the incidence of gout in postmenopausal women [13].

In a study conducted by Lucas et al., it was found that salivary uric acid levels were raised significantly in subjects with acute social stress [14]. Uric acid is therefore implicated in the regulation of neurological physiology in response to psychological stress.

Salivary uric acid levels in metabolic syndrome

Reactive oxygen species and reactive nitrogen species play an important role in signaling and metabolic pathways that have a vital role in the normal physiological functioning of the body. However, a decrease in the antioxidant levels or increase seen in the formation of reactive metabolites can lead to the disruption of homeostasis of the body, which may ultimately lead to oxidative damage. Oxidative stress may leave a serious impact on the viability of the cell and may induce responses on the cellular level, resulting in the death of the cell. Various studies have established a connection between oxidative molecular damage and pathophysiological mechanisms, which are related to severe diseases, including atherosclerosis, diabetes, and neurodegenerative conditions [15]. Oxidative damage may also be linked to the etiology behind inflammatory disorders [16]. Studies have also shown an association between oxidative stress and the aging process [17].

Obesity is a condition that is linked with increased oxidative stress, which can be defined as an increase in a load of free radicals including reactive oxygen species and reactive nitrogen species formed as a result of cellular metabolism. These free radicals are highly active chemically and may lead to the damage of proteins, membranes, and DNA. This increase seen in oxidative stress is also considered to be associated with obesity and type 2 diabetes mellitus [18].

Data from certain studies have shown that the products formed as a result of oxidative stress are responsible for the impairment of insulin-mediated translocation of glucose transporter type 4 (GLUT4) in the myotubes and adipocytes, and lead to the reduction of the transcription of the insulin gene in beta cells and adiponectin in the adipocytes [19].

Measurement of oxidative biomarkers done in the saliva to diagnose various clinical disorders had gained popularity over the last 10 years. As obesity is a chronic state in which diverse pathways, including oxidative stress, endothelial dysfunction, and inflammation, are involved, oxidative biomarkers might prove to be an essential diagnostic tool for obesity when measured in the saliva [19]. In a study conducted by Soukup et al., it was found that salivary uric acid levels were significantly increased in patients with metabolic syndrome, with a p-value of 0.002 [20]. Positive correlations were found between the concentration of uric acid in saliva and diastolic and systolic blood pressure, body mass index, waist circumference, fasting blood glucose, cardiovascular risk factors, and triglycerides.

Uric acid is the end product of purine metabolism, and it adds to the antioxidant capability of saliva and blood [21]. On the other hand, the enzyme responsible for forming uric acid is also responsible for the formation of free radicals with various studies showing uric acid as a culprit that causes pro-inflammatory and pro-oxidant changes [22]. Therefore, there are two schools of thought signifying that uric acid might be an indicator or a causative factor for metabolic syndrome.

Salivary uric acid levels in human immunodeficiency virus

Free radicals or reactive oxygen species are formed whenever there is a reduction in the molecular oxygen/water ratio. They lead to severe damage to DNA, proteins, lipids, and carbohydrates and are also involved in the pathogenesis of various disorders [2]. A line of defense against the reactive oxygen species are the antioxidants that work with the reactive oxygen species in vivo; however, whenever there is an increase in the formation of reactive oxygen species, there is resultant oxidative stress and damage to the cell. This oxidative stress can be internal or external in origin. Infections, medical and oral disorders, and irradiation are a few causes of this oxidative stress. Antioxidants can be found in all body fluids, including saliva [2]. Therefore, saliva is an important factor for maintaining oral health. Uric acid remains the primary predominant antioxidant found in the saliva and accounts for 70%-85% of the total antioxidant capacity of saliva [2]. Uric acid is responsible for scavenging the reactive nitrogen species found in saliva.

Saliva has a normal pH of around 6.7, and it is responsible for regulating the pH of the oral cavity between 6.7 and 7.3 via two methods, the first being the elimination of carbohydrates and acid, which are formed by bacteria, and the second being the neutralization of pH by its buffering action [23]. Hegde et al., in a study conducted on salivary pH and buffering capacity of the saliva in the initial and late stages of the human immunodeficiency virus (HIV) infection, found a reduced pH of saliva and buffering capacity in HIV-positive patients [24]. This decrease was linked to the degree of immunosuppression present in the patient. It is proposed that an increased level of oxidative stress might have a role in the pathogenesis of HIV. In another study conducted by Liu et al., the level of total serum antioxidant capacity of patients with HIV-positive status was found to be lower than that of the control group [2]. In a study conducted by Ahmadi-Motamayel et al., the levels of uric acid in the saliva were analyzed for HIV-positive patients and the control group, and it was concluded that uric acid concentrations in the saliva of patients with HIV were significantly lower when compared with the control group [25].

Salivary uric acid levels in cancer

Several studies have concluded that a rise in the concentration of uric acid may be a marker of oxidative stress and the progression of dangerous medical conditions including cancer of the head and neck [26].

One of the cancers with exponentially high mortality is oral squamous cell carcinoma (OSCC) [27]. Free radicals that are found in the saliva are responsible for oxidative stress that plays a vital role in the pathophysiology of oral squamous cell carcinoma.

As mentioned before, uric acid is responsible for scavenging disease-causing free radicals; hence, a decrease in salivary uric acid might be linked to oral malignancy. In a study conducted by Salian et al., saliva were collected from a total of 50 subjects, comprising 25 subjects with oral squamous cell carcinoma and 25 healthy individuals. A mean uric acid level of 120.7 μmol/L was observed in the patients with oral squamous cell carcinoma, which was reduced significantly when compared with the control group who had a mean uric acid level of 320.0 μmol/L [28]. Nitric oxide concentrations were increased in patients with OSCC. This study strongly indicated a link between salivary uric acid levels and oral cancer, but further studies are required to confirm this association.

The limitation faced by studies involving patients with oral cancer is that the concentration of uric acid levels in the saliva post-radiotherapy is reduced due to hyposalivation.

In a study conducted by Yadav et al. based on the assessment of serum uric acid in leukoplakia, submucous fibrosis, and squamous cell carcinoma, it was concluded that serum uric acid levels were reduced in patients with oral leukoplakia (OL) and OSCC, but they were increased in patients with oral submucous fibrosis (OSMF) when compared with the healthy control [29]. Given the linear association between serum and salivary uric acid, further studies might be carried out to associate salivary uric acid levels with premalignant and malignant conditions of the oral cavity.

In a study conducted by Almadori et al., glutathione and uric acid levels were evaluated in the saliva of patients diagnosed with head and neck squamous cell carcinoma (HNSCC) and a control group, and no statistically significant association was found in the case group with salivary uric acid [30].

In another study conducted by Giebułtowicz et al., salivary uric acid levels were measured in cases of oral cancers and odontogenic cysts, along with healthy controls, and it was concluded that major antioxidants such as salivary uric acid were reduced in the cases of oral cancers [31].

Oral lichen planus (OLP) is one of the important diseases with chronic inflammatory changes. The etiology of this condition remains unknown; however, it has a significant impact on patients’ quality of life. Malignant transformation of oral lichen planus in the form of oral squamous cell carcinoma remains one of the most dangerous complications arising from oral lichen planus. In a study conducted by Darczuk et al., it was concluded that the levels of salivary antioxidants such as uric acid were reduced significantly in patients with oral lichen planus when compared with the control group [32].

Salivary uric acid levels in hyperuricemia

Shibasaki et al. reported the significance of uric acid levels in saliva in patients with hyperuricemia; they also reported an association between serum levels and salivary uric acid [33].

The association between the levels of serum and salivary uric acid was observed in 244 subjects who were divided into two subgroups, one with normal serum uric acid levels and the other with increased serum uric acid levels. The group with normal serum uric acid levels was divided into untreated and treated groups. Uric acid levels in saliva and serum were found to correlate significantly in the untreated group of subjects, with a p-value of less than 0.01. The group of subjects with raised serum uric acid levels was again divided into the treated and untreated groups. The untreated group with increased serum uric acid levels was found to have significantly raised uric acid levels in the saliva and serum when compared with the subjects with normal serum uric acid levels. Another finding was that the uric acid levels in the serum of the untreated group were found to be raised significantly when compared with saliva, with a p-value of less than 0.01. The opposite was found in the treated group, where uric acid levels in saliva were raised significantly compared with the untreated group. This shows that oral intake of drugs has an effect on salivary uric acid levels. This study also suggested a linear association between serum and salivary uric acid levels, which was significant.

Therefore, salivary uric acid can potentially be a substitute for uric acid level determination in blood.

Monitoring of salivary uric acid levels during therapy

It is essential to monitor uric acid levels in patients who receive urate-lowering treatment to be able to select effective drugs and dosages. In a case study by Zhao et al., salivary uric acid levels were monitored in patients on treatment for hyperuricemia instead of serum uric acid levels [34]. In this study, the used drugs were allopurinol and benzbromarone. A decrease in the levels of salivary uric acid from 513 ± 67 μmol/L (619 μmol/L in plasma) to less than 300 μmol/L (360 μmol/L in plasma) was observed, indicating treatment efficacy. However, the ratio of salivary uric acid/uric acid levels in plasma was unaffected by treatment.

Limitations of the salivary uric acid analysis

Despite the cost and noninvasive benefits of salivary uric acid analysis, it has failed to become a popular investigation in various laboratories throughout the world. There is an unfortunate occurrence of interferences that are associated with insufficient volume of the sample of saliva obtained, reduced sensitivity of salivary concentration of uric acid, and early expiration of reagents used in the salivary uric analysis that can affect the reaction and lead to a false-positive or false-negative results [35]. Another limitation of salivary uric acid estimation is the dependence of salivary uric acid on a hydrophilic environment for its antioxidant activity. Also, there is a requirement of ascorbic acid in the plasma to obtain antioxidant activity. Another limitation of the salivary analysis remains the protocol of collection, which is not followed in all centers uniformly. Saliva samples should be collected after a fasting period of eight hours in the morning as the production of saliva is generally increased after having food. Also, the subjects of salivary uric acid estimation should be instructed not to consume food that is high in sugar content and food items containing caffeine, along with abstinence from smoking at least for 30 minutes before sample collection. Any dental treatment should also be avoided within one day before the collection of saliva samples as a dental treatment prior to obtaining saliva might result in contamination with blood [35]. In a study conducted by Kamodyová et al., there was a collection of unstimulated saliva samples from 19 healthy participants three times in a single day, which was done prior to and after brushing of teeth and before and after administering vitamin C (250 mg). It was observed that brushing of teeth and administering vitamin C lead to a decrease in the carbonyl stress and an increase in the antioxidant status of saliva [36].

Therefore, the development of new methods for the determination of salivary uric acid is highly desirable, and further studies are required for the same in order to obtain a standardized method of salivary uric acid analysis.

## Conclusions

The utilization of saliva as a means of sample matrix is increasing in the modern-day era. The estimation of salivary uric acid levels as an important antioxidant can be important in order to diagnose various medical conditions. The determination of salivary uric acid levels is a cost-effective and noninvasive method of investigation that needs to gain more recognition to get standardization worldwide. Further studies are required to explore the full potential of salivary uric acid in modern-day diagnosis.

## References

[1] Greabu M, Battino M, Mohora M (2009). Saliva--a diagnostic window to the body, both in health and in disease. J Med Life.

[2] Liu J, Duan Y (2012). Saliva: a potential media for disease diagnostics and monitoring. Oral Oncol.

[3] Inoue K, Namiki T, Iwasaki Y, Yoshimura Y, Nakazawa H (2003). Determination of uric acid in human saliva by high-performance liquid chromatography with amperometric electrochemical detection. J Chromatogr B Analyt Technol Biomed Life Sci.

[4] Efstathiadou A, Gill D, McGrane F, Quinn T, Dawson J (2019). Genetically determined uric acid and the risk of cardiovascular and neurovascular diseases: a Mendelian randomization study of outcomes investigated in randomized trials. J Am Heart Assoc.

[5] Wang Q, Wen X, Kong J (2020). Recent progress on uric acid detection: a review. Crit Rev Anal Chem.

[6] Püschl IC, Bonde L, Reading IC, Maguire P, Macklon NS, Van Rijn BB (2020). Salivary uric acid as a predictive test of preeclampsia, pregnancy-induced hypertension and preterm delivery: a pilot study. Acta Obstet Gynecol Scand.

[7] Samaranayake L (2007). Saliva as a diagnostic fluid. Int Dent J.

[8] Vernerová A, Kujovská Krčmová L, Melichar B, Švec F (2020). Non-invasive determination of uric acid in human saliva in the diagnosis of serious disorders. Clin Chem Lab Med.

[9] Kim S, Rhee SJ, Song Y, Ahn YM (2020). Comparison of serum uric acid in major depressive disorder and bipolar disorder: a retrospective chart review study. J Korean Med Sci.

[10] Umeki S (1988). Biochemical abnormalities of the serum in anorexia nervosa. J Nerv Ment Dis.

[11] Gupta MA, Kavanaugh-Danelon D (1989). Elevated serum uric acid in eating disorders: a possible index of strenuous physical activity and starvation. International Journal of Eating Disorders.

[12] Giesser R, Goltser-Dubner T, Pevzner D (2020). Elevated salivary uric acid levels among adolescents with eating disorders. Eat Weight Disord.

[13] Hak AE, Choi HK (2008). Menopause, postmenopausal hormone use and serum uric acid levels in US women--the Third National Health and Nutrition Examination Survey. Arthritis Res Ther.

[14] Lucas T, Riis JL, Buchalski Z, Drolet CE, Dawadi A, Granger DA (2020). Reactivity of salivary uric acid in response to social evaluative stress in African Americans. Biol Psychol.

[15] Al-Aubaidy HA, Jelinek HF (2011). Oxidative DNA damage and obesity in type 2 diabetes mellitus. Eur J Endocrinol.

[16] Maddux BA, See W, Lawrence JC Jr, Goldfine AL, Goldfine ID, Evans JL (2001). Protection against oxidative stress-induced insulin resistance in rat L6 muscle cells by mircomolar concentrations of alpha-lipoic acid. Diabetes.

[17] Furukawa S, Fujita T, Shimabukuro M (2004). Increased oxidative stress in obesity and its impact on metabolic syndrome. J Clin Invest.

[18] Lee YH, Wong DT (2009). Saliva: an emerging biofluid for early detection of diseases. Am J Dent.

[19] James PT, Leach R, Kalamara E, Shayeghi M (2001). The worldwide obesity epidemic. Obes Res.

[20] Soukup M, Biesiada I, Henderson A (2012). Salivary uric acid as a noninvasive biomarker of metabolic syndrome. Diabetol Metab Syndr.

[21] Maiuolo J, Oppedisano F, Gratteri S, Muscoli C, Mollace V (2016). Regulation of uric acid metabolism and excretion. Int J Cardiol.

[22] Lippi G, Montagnana M, Franchini M, Favaloro EJ, Targher G (2008). The paradoxical relationship between serum uric acid and cardiovascular disease. Clin Chim Acta.

[23] Carpenter GH (2013). The secretion, components, and properties of saliva. Annu Rev Food Sci Technol.

[24] Hegde MN, Malhotra A, Hegde ND (2013). Salivary pH and buffering capacity in early and late human immunodeficiency virus infection. Dent Res J (Isfahan).

[25] Ahmadi-Motamayel F, Amjad SV, Goodarzi MT, Poorolajal J (2018). Evaluation of salivary uric acid and pH in human immunodeficiency virus infected patients: a historical cohort study. Infect Disord Drug Targets.

[26] Dequanter D, Dok R, Nuyts S Basal oxidative stress ratio of head and neck squamous cell carcinomas correlates with nodal metastatic spread in patients under therapy. Onco Targets Ther.

[27] Johnson DE, Burtness B, Leemans CR, Lui VW, Bauman JE, Grandis JR (2020). Head and neck squamous cell carcinoma. Nat Rev Dis Primers.

[28] Salian V, Demeri F, Kumari S (2015). Estimation of salivary nitric oxide and uric acid levels in oral squamous cell carcinoma and healthy controls. Clin Cancer Investig J.

[29] Yadav K, Patil B, Raheel SA (2020). Serum uric acid levels in patients with oral cancer, leukoplakia and submucous fibrosis: a cross-sectional study. Transl Cancer Res.

[30] Almadori G, Bussu F, Galli J (2007). Salivary glutathione and uric acid levels in patients with head and neck squamous cell carcinoma. Head Neck.

[31] Giebułtowicz J, Wroczyński P, Samolczyk-Wanyura D (2011). Comparison of antioxidant enzymes activity and the concentration of uric acid in the saliva of patients with oral cavity cancer, odontogenic cysts and healthy subjects. J Oral Pathol Med.

[32] Darczuk D, Krzyściak W, Bystrowska B, Kęsek B, Kościelniak D, Chomyszyn-Gajewska M, Kaczmarzyk T (2019). The relationship between the concentration of salivary tyrosine and antioxidants in patients with oral lichen planus. Oxid Med Cell Longev.

[33] Shibasaki K, Kimura M, Ikarashi R, Yamaguchi A, Watanabe T (2012). Uric acid concentration in saliva and its changes with the patients receiving treatment for hyperuricemia. Metabolomics.

[34] Zhao J, Huang Y (2015). Salivary uric acid as a noninvasive biomarker for monitoring the efficacy of urate-lowering therapy in a patient with chronic gouty arthropathy. Clin Chim Acta.

[35] Kamodyová N, Baňasová L, Janšáková K, Koborová I, Tóthová Ľ, Stanko P, Celec P (2015). Blood contamination in saliva: impact on the measurement of salivary oxidative stress markers. Dis Markers.

[36] Kamodyová N, Tóthová L, Celec P (2013). Salivary markers of oxidative stress and antioxidant status: influence of external factors. Dis Markers.

